# The Sleeve Bypass Trial: a multicentre randomized controlled trial comparing the long term outcome of laparoscopic sleeve gastrectomy and gastric bypass for morbid obesity in terms of excess BMI loss percentage and quality of life

**DOI:** 10.1186/s40608-015-0058-0

**Published:** 2015-08-26

**Authors:** L. Ulas Biter, Ralph P. M. Gadiot, Brechtje A. Grotenhuis, Martin Dunkelgrün, Stefanie R. van Mil, Hans J. J. Zengerink, J. Frans Smulders, Guido H. H. Mannaerts

**Affiliations:** Department of Surgery, St. Franciscus Gasthuis, Rotterdam, The Netherlands; Department of Surgery, Catharina Ziekenhuis, Eindhoven, The Netherlands

**Keywords:** Bariatric surgery, Sleeve, Bypass

## Abstract

**Background:**

Obesity is an increasing disease worldwide. Bariatric surgery is the only effective therapy to induce sufficient long-term weight loss for morbidly obese patients. Laparoscopic Roux-en-Y Gastric Bypass (LRYGB) is the gold standard surgical technique. Laparoscopic Sleeve Gastrectomy (LSG) is a new promising bariatric procedure which has the advantage of maintaining an intact gastrointestinal tract. The aim of this study is to evaluate the efficiency of both techniques. Our hypothesis is that LSG has a similar percentage excess BMI loss (%EBMIL) after 5 years compared to LRYGB.

**Methods/Design:**

The Sleeve Bypass Trial is a randomized multicentre clinical trial: patients eligible for bariatric surgery are randomized to either LSG or LRYGB. Patients with a body mass index (BMI) ≥ 40 kg/m^2^ or BMI 35 kg/m^2^ with obesity related comorbidity (T2 DM, sleep apnoea, hypertension) are eligible for randomization. At randomization patients are stratified for centre, sex, T2 DM and BMI ≥ 50 kg/m^2^. A total number of 620 patients will be enrolled and equally (1:1) randomized to both treatment arms. Only surgeons experienced in both operation techniques will participate in the Sleeve Bypass trial. The primary endpoint is the 5-year weight loss (%EBMIL) of LSG and LRYGB. Secondary endpoints are resolution of obesity related comorbidity, complications, revision bariatric surgery and quality of life (QOL) defined in various questionnaires.

**Discussion:**

Long-term %EBMIL between the two treatment strategies used to be in favour of LRYGB, but more recent results throughout the world show similar %EBMIL in both techniques. If weight loss is comparable, obesity-related comorbidity and QOL after bariatric procedures should be taken into account when deciding on which surgical technique is to be preferred for certain subgroups in the future.

**Trial registration:**

Dutch Trial Register: NTR 4741.

## Background

Obesity is a global problem. It induces health risks, diminishes quality of life, psychosocial problems and increases public costs. When patients become morbidly obese, health risks increase rapidly [1]. Morbid obesity is defined as having a body mass index (BMI) ≥ 40 or BMI 35 kg/m^2^ combined with at least one comorbid condition, such as type 2 diabetes (T2 DM), hypertension, dyslipidaemia, or sleep apnoea.

Bariatric surgery is considered the best treatment to realize long-term sufficient weight loss in morbidly obese patients [2–4]. Besides weight loss, it also has profound effects on obesity-related comorbidities, such as T2DM, hypertension and sleep apnoea [5, 6].

Laparoscopic Roux-en-Y gastric bypass (LRYGB) is considered the best surgical option for morbid obesity and as a result is still one of the most frequently performed bariatric procedure worldwide [7, 8]. There are advantages of LRYGB being fully reversible, well documented in terms of early morbidity and long term results known for more than 50 years and often regarded as the gold standard in bariatric and metabolic surgery. Laparoscopic sleeve gastrectomy (LSG) is a relatively new procedure which started as a first stage procedure before a duodenal switch (DS) in super obese patients to reduce treatment related mortality. As weight loss results following LSG were excellent, necessitating a second stage procedure in only a quarter of the patients, LSG is currently used as a single stage procedure in morbidly obese patients. LSG is becoming more popular and is performed in 28 % of bariatric procedures worldwide in 2011 [8]. LSG has some potential advantages; it is a faster and safe procedure where the duodenum is still accessible for endoscopy, there is less dumping due to the pylorus and second stage procedures (LRYGB or BPD-duodenal switch) are standard procedures. The most profound possible disadvantage is that LSG is irreversible. Quality of life (QOL) appears to be similar or even better after LSG compared to LRYGB due to the mentioned advantages [9, 10].

So far results of RCTs with small number of patients and short-term outcome have been published only. In these studies the percentage excess BMI loss (%EBMIL) appeared to be equal for both techniques [9, 11–16]. However, concerns remain that the sleeve will lose its effect in time, resulting in a decline of weight loss and reappearance of obesity-related comorbidity [17].

## Methods/Design

### Study objective

The primary aim of this multicentre study is to compare LSG with standard LRYGB in terms of percentage excess BMI loss (%EBMIL) as well as in secondary outcomes (e.g. QOL, resolution of obesity-related-comorbidity, complications and revision bariatric surgery due to insufficient weight loss or GERD). In order to determine the area of indication for the LSG in the field of bariatric surgery patients in this study are also stratified at randomisation for sex, T2 DM and a BMI below or above 50 kg/m^2^.

### Study design

**Fig. 1:**
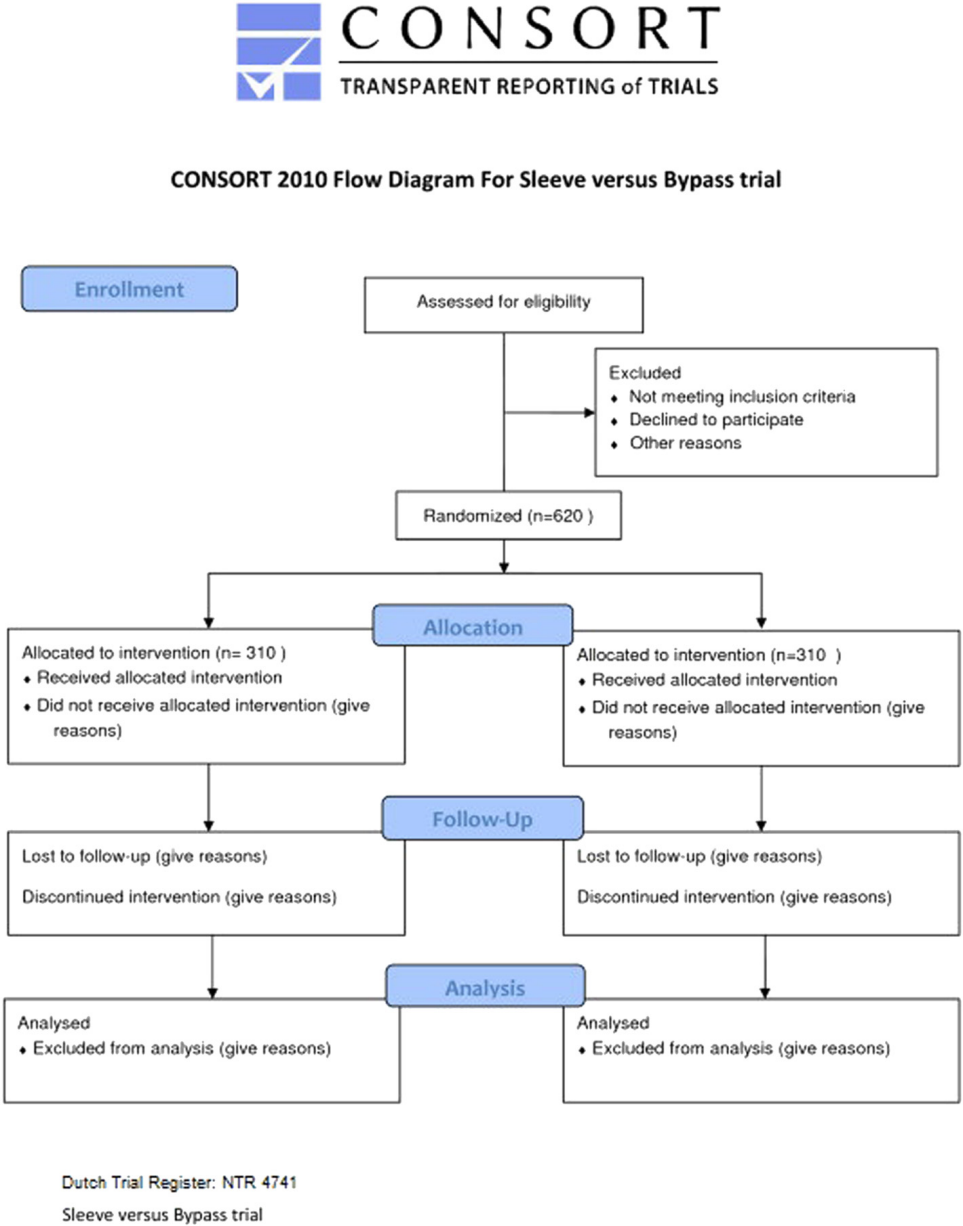
CONSORT flowchart

The sleeve versus bypass trial is a randomized multi-centre trial, in which two bariatric medical centres are participating. The study started on November 24th 2012 and the duration of the inclusion will be approximately 3 years. The study compares the %EBMIL of LSG and LRYGB after five years (primary endpoint). Patients with morbid obesity with a BMI ≥ 40 kg/m2 or BMI 35 kg/m2 with obesity related comorbidity (T2 DM, sleep apnoea, hypertension) are eligible for randomization. In total 620 morbidly obese patients will be included in the study. Approval of the medical ethical committee of both participating centres, “Toetsingcommissie Wetenschappelijk Onderzoek Rotterdam” and “Medisch Ethische Toetsingcommissie Catharina Ziekenhuis Eindhoven” was obtained. After approval the trial is accepted and registered in the Dutch trial register. The complete route of patient inclusion and randomisation is depicted in Fig. 1.

Patients will be randomized, using a randomization website to one of the following bariatric procedures:

1. Laparoscopic sleeve gastrectomy (LSG)
  The LSG is performed by a full mobilisation of the greater curvature and the posterior stomach, followed by stapling calibrated over a 34 french boogie, starting 2–3 cm’s from the pylorus. The used technique is described in 2012 and proven to be effective and safe [18].
2. Laparoscopic Roux-and-Y gastric bypass (LRYGB)
  The LRYGB is performed with the antecolic linear technique. A small 4 cm long pouch is calibrated over a 34 french boogie and 3 cm linear gastroenterostomy is realized. The measured biliopancreatic limb is 60 cm and the alimentary limb is 150 cm. The omentum can be divided at the surgeons’ discretion. Both mesenteric defects are closed with clips [19].

### Patient selection

All morbidly obese patients who have been approved for bariatric surgery by the preoperative multidisciplinary team can be included in the sleeve versus bypass trial. Informed consent is mandatory. The criteria for bariatric surgery are age 18–60 years, BMI > 40 kg/m^2^, or BMI > 35 kg/m^2^ with obesity-related comorbidity (such as T2 DM, hypertension, hypercholesterolemia, severe arthrosis and OSAS) for more than 3 years, conservative therapy (preferably under the guidance of a physician or self-help group) that has failed or showed only transient results, completion of psychological screening, excluding patients with psychiatric and psychological disorders, written informed consent and willingness to conclude the lifelong follow up program after surgery.

Exclusion criteria for this study are: a history of diagnosed symptomatic gastro oesophageal reflux disease (GERD) [20], or a diagnosed hiatal hernia with symptoms. There is no routine gastroscopy prior to surgery in asymptomatic patients. Other exclusion criteria are severe sweet eating [21], prior bariatric surgery, prior major abdominal surgery (such as colonic resection, abdominal sepsis, aortic surgery, which might jeopardise the technical feasibility of LSG or LRYGB) and the inability of reading or understanding the questionnaires.

### Hypothesis

Our hypothesis is that LSG has a comparable %EBMIL after 5 years when compared to LRYGB [22–26]. Even if LSG is marginally less effective compared to LRYGB in terms of long term %EBMIL, QOL might be better from a dietary and long term complications point of view [27-29].

### Study questions

Primary Question: Does LSG result in a similar long term %EBMIL after a follow up period of 5 years when compared to the LRYGB?

Secondary Question: Is the QOL better after a LSG compared with the LRYGB?

Other secondary endpoints: improvement in obesity induced co-morbidity, complication rate, readmission and reoperation rate, revision surgery rate, operating time and duration of hospital stay and the predictive value of sweet eating habit inventoried by the Dutch Sweet Eating Questionnaire (DSEQ) [21].

### Surgical intervention

All patients are treated in a fast-track protocol [30] and discharged 1 day after surgery. Antithrombotic prophylaxes (Fragmin® 5000 u/day) and a fluid diet is continued for 2 weeks postoperatively. All patients are prescribed proton pump inhibitors for 6 weeks after surgery. Follow up of patients will be performed in an outpatient clinical setting [Table 1].

**Table 1:** Follow up after surgery

Follow up	History/Examination	Weight/Complications	Questionnaires^a^
1 Week	Obesity nurse	Obesity nurse	
6 Weeks	Surgeon	Surgeon	Obesity nurse
3 Months	Endocrinologist^b^	Endocrinologist^b^	
6 Months	Endocrinologist^b^	Endocrinologist^b^	Obesity nurse
9 Months	Endocrinologist^b^	Endocrinologist^b^	
12 Months	Surgeon	Surgeon	Obesity nurse
Annualy	Endocrinologist^b^	Endocrinologist^b^	Obesity nurse

^a^Changes in health status over time are measured using generic and disease specific quality of life questionnaires (Euro-Qol 5D, Short Form 36 (SF-36) and the Gastro-Intestinal Quality of Life Index (Qiqli)) on admission and after 6, 12 months and then annually. Obesity related quality of life questionnaires (the Moorehead-Ardelt II) and the Bariatric Analysis and Reporting Outcome System (BAROS) score are measured at the same time-intervals. Sweet eating will be established with the Dutch Sweet Eating Questionnaire (DSEQ)

^b^Every visit to the endocrinologist is combined with extensive laboratory testing

The patient is called by the obesity nurse in the first week. Outpatient clinic visits are scheduled at 6 weeks, 3, 6 and 12 months and then once annually. The standard follow up [Table 1] is combined with extra visits in case of complaints or possible complications with a 24 h access to the bariatric surgery department. The complications are scored at every consultation using the “Clavien-Dindo classification” to score the severity [31].

All participating surgeons are experienced bariatric surgeons that have performed at least 150 LSG and 150 LRYGB and work in bariatric centres of excellence that perform over 500 cases per year.

#### Laparoscopic sleeve gastrectomy

Two different known approaches have been described in the literature [32, 33]: the stapling first technique or the full mobilisation of the greater gastric curvature first technique. As a tight gastric sleeve, which is important for an optimal weight loss result, seems to be realised best by the gastric mobilisation first technique [18] this is the only LSG technique used for this study.

1. Starting
  After achieving pneumoperitoneum, with a Veress needle in the left upper quadrant, five trocars are placed according to the surgeons preference.
2. Mobilization
  The greater curvature is dissected downward to 2–3 cm proximal to the pylorus. Next the greater curve is further mobilized up to the angle of His, visualizing the complete left diaphragm crus. While mobilizing the greater curve all gastro-pancreatic adhesions at the dorsal smaller gastric curve are also released in upward direction towards the angle of His. This step is important in order to be able to realize a small gastric sleeve without redundant fundus at the dorsal gastric wall.
3. Stapling
  A linear 60 mm endoscopic stapler is introduced through the right trocar and fired at the antrum, leaving just enough space for a gastric boogie to pass. Next an orally introduced gastric tube is inserted up to the pylorus. This gastric tube is 34 French (Fr) in diameter as it is important to create a small gastric sleeve. A tight sleeve is realised by firing another 4–5 60 mm long linear staple lines using the same trocar site. The stapler is placed tight to the tube while stretching the greater curve. Each time after placing the stapler the gastric tube is moved by the anaesthesiologist under laparoscopic vision in order to ensure that the stapler has not been placed on the tube itself. The last stapler is fired lateral to Belsy’s fat leaving the oesophagus unharmed.
4. Finishing
  Next, the left trocar site is dilated and the resected stomach is removed. Subsequently, this trocar site is closed at fascia level. The gastric tube is removed by the anaesthesiologist under laparoscopic vision. Staple line bleeding is controlled by clips and when necessary with sutures. Only in case of bleeding, stapling problems, or other difficulty, a drain is placed along the sleeve gastrectomy. Over-sewing of the staple line is not routinely performed. Next, the trocars are removed under sight and the skin is closed.

#### Laparoscopic Roux-en-Y gastric bypass

1. Starting
  After achieving pneumoperitoneum, with a Veress needle in the left upper quadrant, five trocars are placed according to the surgeons preference.
2. Gastric pouch creation
  At the gastro oesophageal junction Belsy’s fat is released from the left diaphragm crus.
  Next, at the lesser curve, approximately 4 cm below the angle of His, the minor omentum is released from the lesser gastric curve until an opening is created in the omental burse. A 60 mm linear endostapler is used to transect the lesser curve horizontally. Then, the gastro-pancreatic adhesions are released and 2 more 60 mm linear endostaplers are fired in vertical direction along a calibrating 34 Fr gastric tube that has been inserted transorally. Thus a small and calibrated gastric pouch is created.
3. Omental split
  In order to make the pathway of the small bowel towards the gastric pouch shorter the greater omentum can be split vertically.
4. Creation of gastro-enterostomy
  The small bowel is brought upward towards the gastric pouch at a length of 60 cm from the point of Treitz. At this point a small defect is created in the small bowel as well as in the left caudal corner of the pouch. A linear endostapler is fired at 3 cm’s length. The anastomosis is closed by laparoscopic suturing. The gastro-enterostomy is tested by methylene blue injected through a gastric tube. If the test indicates leakage, this point is sutured and the methylene blue test is repeated.
5. Creation of entero-enterostomy
  A linear stapler is fired and the remaining defect is sutured creating an entero-enterostomy between the small bowel proximal to the gastro-enterostomy and the small bowel 150 cm distally from the gastro-enterostomy.
6. Finishing
  Petersen’s space and the mesenteric defect at the entero-enterostomy are closed by a laparoscopic clipping device [19]. Staple line bleeding is controlled by clips and when necessary with sutures. The trocars are removed under sight and the skin is closed.

#### Escape surgery

If it is technically impossible to perform an LRYGB during surgery, it is allowed to perform a LSG. Moreover, if there are technical problems during LSG e.g. creating an occlusion of the sleeve or stapling of the oral tube, conversion to LRYGB surgery is allowed. The statistical analysis will be according to the intention-to-treat principle and a per protocol analysis.

### Outcome measures

#### Primary endpoint

Sustainable weight loss. The amount of weight loss is expressed as percentage excess BMI loss (%EBMIL), and calculated with the formula %EBMIL = (pre-operative BMI – current BMI)/(pre-operative BMI – ideal BMI) × 100 %. For this formula a BMI of 25 kg/m^2^ was taken as the upper limit of normal, i.e. the ideal BMI.

#### Secondary endpoints

To evaluate operating time, duration of hospital stay, intra-operative and post-operative in-hospital mortality and morbidity following LSG or LRYGB. Morbidity is defined as re-operations, re-interventions, re-admissions and serious adverse events. Morbidity is also defined as major (anastomotic leakage, major peroperative blood loss due to splenic or vascular haemorrhage, pulmonary embolism, intra-abdominal abscess and intra-abdominal hematoma) or minor (wound infection, urinary tract infection and anastomotic stenosis). Moreover, the rate of extra outpatient and emergency room visits because of complaints following LSG or LRYGB are evaluated.

Preoperatively all patients are assessed by an multidisciplinary team consisting of at least surgeons, endocrinologists, physiotherapists, nutritionists, and psychiatrists.

The endocrinologist defines the comorbidities using the definitions from common international guidelines [34] for hypertension, dyslipidaemia, T2 DM, obstructive sleep apnoea syndrome (OSAS) and joint pain. The surgeon defines gastro oesophageal reflux disease (GERD) as need for proton pump inhibitor (PPI) agents and/or esophagitis diagnosed on endoscopy. To evaluate the biochemical changes following LRYGB or LSG in the first year extensive laboratory tests are obtained every 3 months. This is continued in the yearly visits which are common practice in our clinics. If patients fail to attend follow up they will be contacted by telephone to motivate them to return to the follow up or the patient’s general practitioner will be contacted in order to minimize the lost-to-follow up. To evaluate remission or improvements of the various comorbidities the use of medication is documented and adjusted where necessary during each visit. The remission of a co-morbidity was defined when patients no longer needed a drug therapy and had normal blood pressure. In T2 DM remission was defined as a normal fasting glucose, without medication and a glycosylated haemoglobin (HbA1c) of <6 %. Improvement was defined as changing from insulin to oral antidiabetic drugs or lowering the dose or number of drugs needed.

To evaluate QOL multiple questionnaires are used. QOL was objectified by the asthma control questionnaire (ACQ), the reflux disease questionnaire (Gerd-Q), the Bariatric Analysis and Reporting Outcome System (BAROS) score, the Gastro-intestinal Quality of life Index (GIQLI), EuroQol-5D (EQ-5D), Short-form 36 (SF-36) before and following LSG or LRYGB.

To evaluate the predictive value of the Sweet eating inventoried by the Dutch Sweet Eating Questionnaire (DSEQ) [17].

To evaluate the need for revision surgery (need to perform an additional bariatric procedure after the performed surgery) as a result of insufficient weight loss or medical complaints within 5 years following the primary bariatric procedure (LSG or LRYGB). Insufficient weight loss is defined with the Reinhold criteria (modified by Christou and Biron).

All data will be collected in a digital patient form in order to ensure completeness of data.

### Data and safety monitoring committee

To ensure patient safety and study integrity, an independent data and safety monitoring committee is established, which will evaluate the progress of the trial and will examine safety parameters at regular intervals of 100 included patients. All involved physicians need to report any potential adverse events encountered in this study and these potential adverse events will be discussed with the monitor committee. The monitoring committee can request a full report in order to discuss a specific adverse event and this report will be sent to the central ethics committee and all involved physicians.

All deceased patients will be evaluated by the safety committee for cause of death and possible trial related serious adverse events. All deaths will be reported to the central ethics committee and the local ethics board. The DSMB consists of an epidemiologist/statistician, an independent surgeon and an independent endocrinologist.

### Power calculation

The data of the randomized subjects are analysed according to the intention to treat principle. Follow up will be completed until 5 years after the operation.

For the sample size determination the used hypothesis for the study are:

- H0: mean %EBMIL (LSG) = mean %EBMIL (LRYGB)
- H1: mean %EBMIL (LSG) ≠ mean %EBMIL (LRYGB)

To be able to reject the null hypothesis that mean %EBMIL after LSG treatment equals mean %EBMIL after LRYGB, at least 2×294 analysable patients have to be included (mean (SD) %EBMIL is 68.59 (25.88) after LSG and 62.60 (25.88) after LRYGB; type I error: 0.05 (two sided), type II error = 0.20 (power = 80 %), randomization ratio 1:1). Considering a dropout rate of 5 %, the sample size is estimated to be 2×294/.95 = 2×310 = 620.

The %EBMIL data of LSG and LRYGB are obtained from the meta analysis of Garb *et al.* [35]. The standard deviation of LRGYB is assumed to be equal to the standard deviation of LSG.

## Discussion

Laparoscopic sleeve gastrectomy (LSG) is a relative new procedure which was first described by Marceau and Hess in the 1990s as a part of the BPD duodenal switch and LSG is later popularized by Regan and Gagner as a first stage procedure prior to a duodenal switch (DS) in super obese patients to reduce treatment related mortality [32]. The weight loss results following LSG were excellent, resulting in a second stage procedure in only a quarter of the patients. Therefore, LSG is currently used as a single stage procedure in morbidly obese patients resulting in a percentage excess BMI loss (%EBMIL) ranging between 50 % and 83 % and having a favourable impact on comorbidities [18–22]. In comparison with LRYGB, LSG is technically more simple and faster to perform. However, currently long term results of LSG are limited compared to LRYGB. Another benefit of LSG is the relatively normal dietary options for the patients, potentially improving QOL in comparison with LRYGB. LRYGB associated dumping syndrome, caused by large particles and carbohydrates directly entering the small intestine, has been reported [23, 24]. Moreover, patients treated by LSG have less nutritional deficits compared to LRYGB [25]. Another benefit of LSG is that, as the normal gastrointestinal tract is preserved, diagnostic and therapeutic interventions such as gastroscopy and ERCP are still possible. Especially the inability to perform an ERCP can be a problem after LRYGB as many morbidly obese patients suffer from bile stone related problems following bariatric surgery as a result of weight loss and concurrent cholecystectomy has been abandoned since this can increase perioperative risks and complications. Furthermore, LSG can easily be converted to LRYGB, BPD-DS or an omega loop bypass depending on the patient characteristics and the preferences of the surgeon in case of insufficient weight reduction. Moreover LSG results in a better QOL than LRYGB [9].

Currently, only few randomized controlled trials have compared LSG and LRYGB [11, 19]. However, these studies described small study groups and did not report long-term results. Although their conclusions lack hard evidence to determine in which patients these two different techniques should be performed, these studies seem to show a small short term beneficial result for LSG.

This study will contribute to the evidence of the benefits of LSG versus the standard LRYGB.
